# Violence in Dental Operations

**Published:** 1867-01

**Authors:** J. P. H. Brown


### VIOLENCE IN DENTAL OPERATIONS.

BY J. P. H. BROWN.

It is a professional axiom, that every dental operation should be performed with *thoroughness and skill.* If, in order to execute any operation upon the teeth, or adjacent parts, in a faithful and skillful manner, it becomes necessary to inflict pain, it should be done. But in doing so, the constitutional diathesis of the patient, as well as the susceptibility of the parts operated on to morbid action, must never be lost sight of. When these idiosyncracies of of the patient are not taken into account, our treatment may amount to violence as the following case will show:

I have now under treatment a patient who, three years ago, had his upper central incisors separated with rubber, by a dentist preparatory to filling. To use the patient's language, his dentist "*violently* forced it between the teeth and into the gum." The result of this violence was inflammation of the alveolo-dental periosteum of the right central incisor, and from thence gradually extended as far back as the first molar. The periosteal inflammation terminated in necrosis of the alveolar processes of the right central and lateral incisor, and of the right cuspid and bicuspids, and also of a considerable portion of the jaw, and of a part of the palatine bone. The sequestrum has not yet separated from the body of the jaw. The patient has what is termed the strumus diathesis.

Had the dentist, in this case, possessed a sufficient amount of medico-dental knowledge, he would have introduced his rubber, not with violence, but with a due regard to the susceptibility of the parts to diseased action. The file, rubber, wood, cotton-wedges, &c., are all most excellent for separating teeth when rightly applied to just the right case. *Routine* in the practice of dentistry is just as reprehensible as it is in the practice of medicine.

